# *Shewanella chilikensis* MG22 isolated from tannery site for malachite green decolorization in microbial fuel cell: a proposed solution for recirculating aquaculture system (RAS)

**DOI:** 10.1186/s12934-023-02152-9

**Published:** 2023-08-01

**Authors:** Abanaoub Efraim, Mai Saeed, Mariam Ahmed Elbaz, Mohamed Alaa, Noran Ahmed, Rana Adel, Yara Hazem, Einas Elshatoury, Ola M. Gomaa

**Affiliations:** 1 Microbiology Department, Applied Biotechnology Section, Faculty of Science, Ain Shams University, Cairo, Egypt; 2 Radiation Microbiology Department, National Center for Radiation Research and Technology (NCRRT), Egyptian Atomic Energy Authority (EAEA), Cairo, Egypt

**Keywords:** Microbial fuel cell, Malachite green, *Shewanella chilikensis*, Aquaculture, Decolorization, Recirculating aquaculture system (RAS)

## Abstract

Malachite Green (MG) dye of the triphenylmethane group is a toxic compound used in the aquaculture industry as an antifungal agent, however, it can accumulate in fish and pose toxicity. The present work aims to remove MG in Microbial Fuel Cell (MFC) as a sustainable and eco-friendly solution. Out of six samples, the highest malachite green degradation was obtained by a sample obtained from Robiki tannery site in agar plates in 24 h at 37 °C. Robiki sample was used to inoculate the anodic chamber in Microbial Fuel cell, the resulting average electricity production was 195.76 mV for two weeks. The decolorization average was almost 88%. The predominant bacteria responsible for MG decolorization and electricity production were identified using 16S rRNA as *Shewanella chilikensis* strain MG22 (Accession no. OP795826) and formed a heavy biofilm on the anode. At the end of the decolorization process, MG was added again for re-use of water. The results showed efficiency for re-use 3 times. To ensure the sterility of treated water for re-use, both UV and filter sterilization were used, the latter proved more efficient. The obtained results are promising, MFC can be used as recirculating aquaculture system (RAS). The same aquaculture water can be treated multiple times which provides a sustainable solution for water conservation.

**Graphical Abstract:**

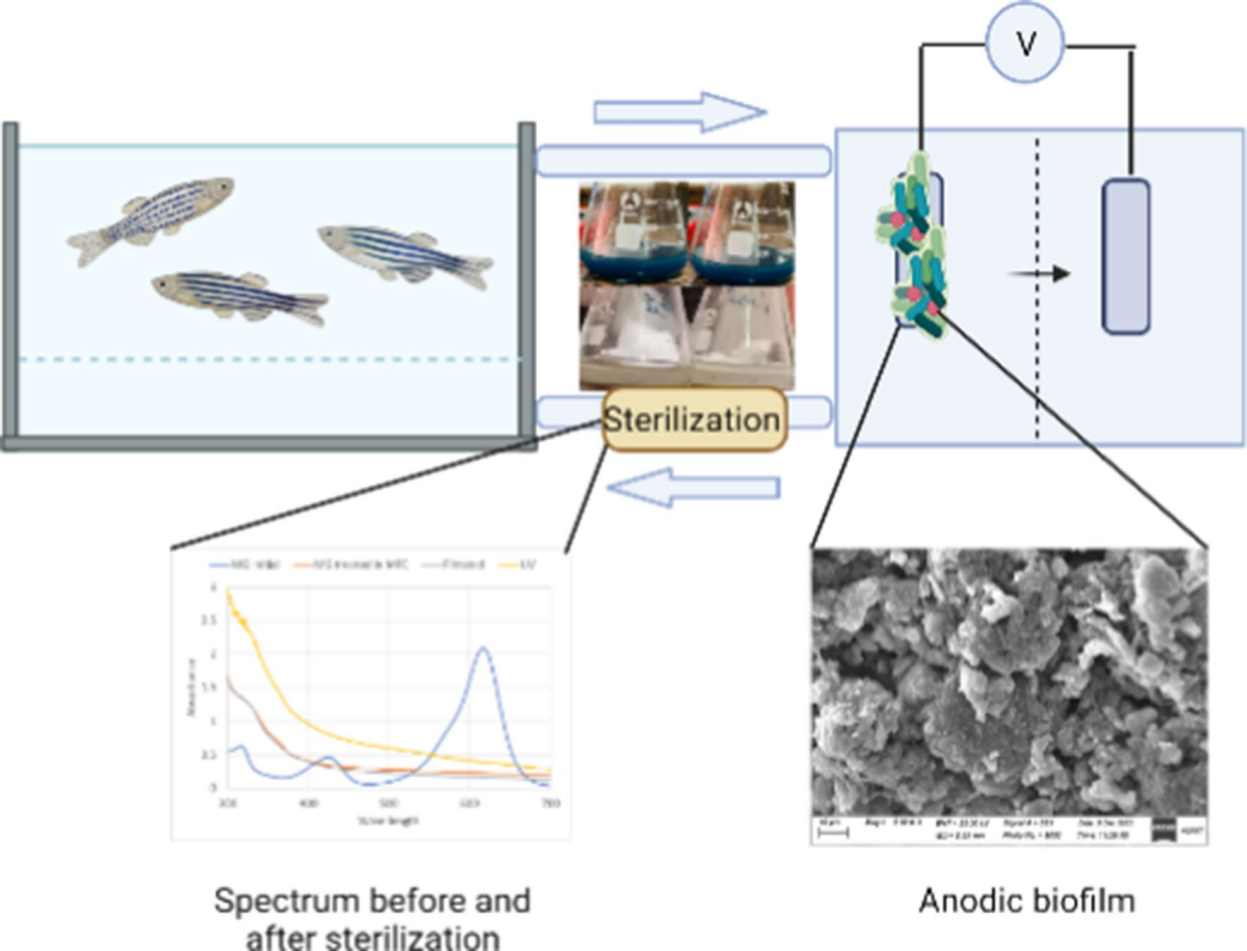

**Supplementary Information:**

The online version contains supplementary material available at 10.1186/s12934-023-02152-9.

## Introduction

Malachite green (MG, C_23_H_25_C_1_N_2_) is an organic dye that belongs to the triaryl methane dye class. Despite it being banned in many developed countries, it is still used due to its low cost, effective antimicrobial activity, and strong dying properties. It is demanded in different fields such as pharmaceutical industry, textile industry, and is also used in aquaculture. Cracknell [5] reported that about 0.1 mg/L is needed in aquaculture and is used as antiprotozoal and antifungal medication for fish. Sutili et al. [31] reviewed the different concentrations required, the report mentioned 3–5 mg/L to control egg fish saprolegeniosis, 0.25 mg/L to kill mycelia on fish and 0.15 mg/L to disinfect fish larvae. Malachite green’s solubility and stability in water prevent its biodegradation rendering it among the persistent compounds [28]. Malachite green persistence in aquaculture leads to different carcinogenic, mutagenic, and teratogenic effects in humans besides its high acute toxicity in fish [30]. Since aquaculture is considered a very important source of protein in many developing countries, the need for a sustainable approach is needed to ensure good quality water and hence good quality fish that is appropriate for human consumption. Several methods were reported to remove malachite green from water, such as purification by extraction filtration strategy [16], photocatalytic [8], integrated biochar adsorption and phycoremediation [18], ultrasonic assisted electrochemical degradation [22] and Microbial fuel Cells [2].

Microbial fuel cells (MFCs) convert chemical energy to electrical energy and can remove different pollutants from waste streams and aquaculture sediments using electroactive bacteria [15, 36]. Electroactive bacteria are known to have two main mechanisms by which electrons are harnessed, (1) direct electron transfer (DET) where electrons move inward or outward via direct contact with the electrode using nanowires or biofilm formation and (2) mediated electron transfer (MET) by which electrons move via an electron shuttle compound. Several gram-positive and gram-negative bacteria were reported to have electroactivity and electroactive enhancing strategies were reviewed [1, 9]. The use of MFC in dye decolorization has been proven to be efficient [13, 33, 34]. The use of MFC for malachite green decolorization and bioenergy production in MFC has been rarely reported was rarely reported. The only reported research was published by [3] and [2] using shiitake mushrooms. Extremophiles were found to possess electrochemical activity, reviews on acidophiles, psychropiles, xerophiles, alkaliphiles and piezophiles were reported by Dopson et al. [6], while a review on thermophiles and halophiles was reported by Shrestha et al. [26]. The studies link the electroactive activity of extremophiles to their ability to live under extreme conditions and that modifications to their membrane structure or physiological or molecular makeup play a role in their electroactive mechanisms. Moreover, microorganisms isolated from harsh extreme habitats are known to perform biodegradation due to their ability to perform enzymatic and biocatalytic activities [27]. Therefore, isolation from an extreme environment would ensure that microbial growth would not be affected by the presence of pollutants such as malachite green dye or any other present contaminant. From this standpoint, the aim of the present work is to (1) test the decolorization of malachite green in MFC, characterize the predominant decolorizing electroactive bacterial strain responsible for malachite green decolorization, and (2) test multiple decolorizing cycles in MFC for possible integration in Recirculating Aquaculture System (RAS).

## Materials and methods

### Sample collection

Six different samples were collected to isolate malachite green degrading bacteria according to [23]. (1) Textile effluent sample was collected in a 500 ml sterilized plastic bottle from the drainage canal of one place from Robiki leather city(tannery factory)—Badr City and was named as Robiki sample, (2) Fish water sample was collected in a 500 ml sterilized plastic bottles from fish aquaculture tank, (3) Salt samples were collected in 500 ml sterilized plastic bottles from the salt mountain from salt evaporation ponds at Port Fouad, (4) Industrial exchange sample was collected in 500 ml sterilized plastic bottle from drainage canal behind factories at Amriya area at Alexandria, (5) Nile water Sample was collected in a 500 ml sterilized plastic bottle from the Nile under Qasr El Nil Bridge at Cairo, and (6) Mud sample was collected in a 500 ml sterilized plastic bottle from a random underground hole under anaerobic conditions at Cairo and was named as: Mud sample. All samples were stored at 4 °C and transferred to the lab to isolate malachite green dye-degrading bacteria.

### Dyes, chemicals, and microbiological media

Triphenylmethane dye malachite green (MG) was purchased from the local market. Dye degrading mineral salt (MS) medium was prepared by adding the following components (g/L): K_2_HPO_4_ (2), (NH_4_)SO_4_ (0.5), KH_2_PO_4_ (0.2) and MgSO_4_ (0.05) [23], Minimal salt media (g/L): NH_4_Cl (0.46), KCl (0.225), MgSO_4_.7H_2_O (0.117), NaH_2_PO_4_ (2.5), Na_2_HPO_4_ (4.11), (NH_4_)_2_ SO_4_ (0.225) and the trace element (TE) solution was prepared by adding the following components (g/L): FeSO_4_ (0.4), MnSO_4_ (0.4), ZnSO_4_ (0.2), CuSO_4_ (0.04), KI (0.3), Na_2_MoO_4_ (0.05) and CoCl_2_ (0.04).

### Screening of malachite green decolorizing consortia

To test if the bacteria collected can tolerate malachite green dye and degrade it, samples were cultivated in malachite green and were inoculated on nutrient agar medium to grow. The stock solution of malachite green dye with concentration 5 mg/ml. The first screening was performed using agar plates, 0.4 µg/ml of malachite green dye was added to nutrient-molten agar and plated, and 100 µl of each sample was added using the spread plate method technique under aseptic conditions [25]. The plates were incubated in the dark at 37 °C for 24 h. The grown bacteria at this concentration were selected and spread on a higher concentration of malachite green dye by 10 folds (4 µg/ml). Plates were incubated in the dark at 37 °C for 24 h. Grown bacteria at this concentration were further cultured at a higher concentration of malachite green dye (40 µg/ml). The plates were incubated in the dark at 37 °C for 24 h. Finally, the bacteria growing on 40 µg/ml malachite green dye plates were streaked again on the same malachite green dye concentration and were left to incubate in dark at 37 °C for 24 h.

## Malachite green decolorization in batch cultures

MG decolorization was monitored using a spectrophotometer Unico UV-2000. The absorbance was measured at 620 nm by the same spectrophotometer.

$$ {\text{Decolorization }}\left( \% \right)\, = \,(I\, - \,F)/I\, \times \,{1}00 $$

where I: Initial absorbance and F: Final absorbance. To estimate the optimum conditions for these consortia, 4 tests were done for each sample which showed the ability to grow on the highest concentration of malachite green dye (concentration 40 µg/ml) using Robiki sample. Four 250 ml flasks were used for each sample, aerobic with no glucose supplementation, aerobic with 1% glucose supplementation, anaerobic with no glucose supplementation, and anaerobic with 1% glucose supplementation. Cultures were incubated in the dark to avoid photodegradation. Absorbance was recorded at 0, 24, 48, 72, 96, and 120 h.

### Microbial fuel cell set-up and operation

The sample was enriched in nutrient broth and incubated at 37 °C for 24 h. An open circuit double chamber MFC was constructed with Proton Exchange Membrane as the separator as mentioned by [10]. The anodic chamber contained 75% minimal salt media, vitamin mixture, trace mineral solution 1ml (1%), 500 mg/L casein, and 2.2 g/L sodium pyruvate (pH 7) and 20% of 24 h enriched Robiki microbial consortium. Malachite green dye was added at a concentration of 40 µg/ml. The cathodic chamber contained 80% working volume of 0.1 M copper sulfate dissolved in 50 mM sodium phosphate buffer (pH 7). Voltage was recorded periodically and decolorization assay was performed as previously mentioned using a UV spectrophotometer Schimadzu UV 2100.

### Identification of predominant bacteria

At the end of the MFC operation, a sample was plated on LB agar plate. The predominant isolate with malachite green decolorizing ability was chosen for 16S rRNA phylogenetic identification. DNA of a 24 h culture was extracted in 1ml TE buffer (pH8). The cell suspension was boiled for 10 min to release DNA then chilled on ice for 10 min. The suspension was centrifuged at 10,000 rpm for 5 min. About 50 ng of DNA template was added to 45 µl of PCR reaction solution (Macro Gen) using the following primer sets 27–8 GAGTTTGATCCTGGCTCAG and 1492 GGTTACCTTGTTACGA [7]. The amplification was performed as follows: 35 amplification cycles at 94 °C for 45 s, 55 °C for 60 s, and 72 °C for 60 s. An aliquot of 5–15 μl of PCR reaction products was electrophoresed on a 1% agarose gel containing ethidium bromide (10 mg/ml in dH_2_0) and the DNA bands were visualized under the UV light. The amplified PCR products were submitted to Solgent Co Ltd (South Korea) for purification and sequencing. The resulting sequences were trimmed and assembled in Geneious software (Biomatters). The sequence was compared to the NCBI nucleotide database (https://blast.ncbi.nlm.nih.gov/Blast.cgi) and the phylogenetic tree of the strain (accession number: OP795826) was constructed using the neighbor-joining method using www.phylogeny.fr free database.

### Multiple re-uses of MFC

The MFC system was kept running and MG was added 3 times, each time started after decolorization. Samples were drawn and assayed using UV–vis spectrophotometer as previously mentioned.

### Scanning electron microscopy (SEM)

Bacterial biofilm was observed using Scanning Electron Microscopy (SEM). Images of the anode with biofilm growth were captured using a Zeiss evo15 scanning electron microscope (Germany). Carbon electrodes were placed on brass stubs using double-sided adhesive tape. The images were captured at magnifications of 250 and 2000 X using an electron beam high voltage of 20 kV.

### Sterilization of treated wastewater

For safe reuse of the treated wastewater, a sterilization step was performed using UV and filter sterilization prior to its recirculation into the aquaculture system again. After decolorization, samples were subjected to (1) exposure to a UV lamp at 254 nm for 30 min at a 10 cm distance and (2) filter sterilization using 0.2 µm. A UV–Visible spectrum assay was performed using a spectrophotometer and data was plotted before and after UV sterilization and filtration.

## Results and discussion

### Sample collection and screening

Several bacterial species have been shown to degrade dyes effectively. Microbial decolorization is both environmentally and economically advantageous. The criteria used for selecting bacteria in the present experiment was to cultivate samples collected from extreme environments and test their ability to grow and degrade high concentrations of malachite green dye that match the concentration used in Aquacultures. The use of extremophiles for dye decolorization has been studied before since the bacteria shows cross tolerance to different stress and harbors an arsenal of enzymes that could tolerate extreme conditions of textile effluent [19]. After inducing and growing bacteria on MG of concentration 0.4 µg/ml by using Mud sample, the Nile sample, Alexandria sample, Robiki sample, Aquaculture sample and Solid salt sample. But it was observed that the samples: Alexandria, Nile, Aquaculture and Salt samples’ colonies were few, but the remaining samples (Robiki and Mud samples) showed a variety of colonies. Because of their bright and vibrant coloration, these dyes are easily visible. The mud sample and Robiki sample, which produced many colonies on the plate that degrade malachite green and reduce its color, were grown on the higher concentration of MG (4 µg/ml). Growing Robiki and Mud samples on higher concentrations of MG (40 µg/ml), Robiki samples showed full visual degradation of MG dye, on the other hand, Mud samples showed bioaccumulation of MG dye as shown in Table 1. We chose Robiki sample because of the obvious decolorization of the agar plate as compared to Mud sample that showed an accumulation of green color on the colonies (S1). This is in accordance with Shukla and Singh [27] who stated that microorganisms growing in harsh habitats such as extreme temperatures, salinity, alkalinity, or unavailability of nutrients, are known to perform biodegradation because they possess an arsenal of enzymatic and biocatalytic activities. Bacteria isolated from textile effluent were specifically reported to degrade dyes [23]. Therefore, Robiki sample, considered both an extreme habitat and textile effluent, was chosen for further study.

**Table 1:** Screening of malachite green decolorization using different bacterial consortia

Sample	0.4 µg/ml	4 µg/ml	40 µg/ml
Robeki	+	+	+
Fish farm	+	−	−
Salt pond	+	−	−
Industrial plant	+	−	−
River Nile	+	−	−
Mud sample	+	+	+

### Effect of aeration and glucose on malachite green decolorization

**Fig. 1:**
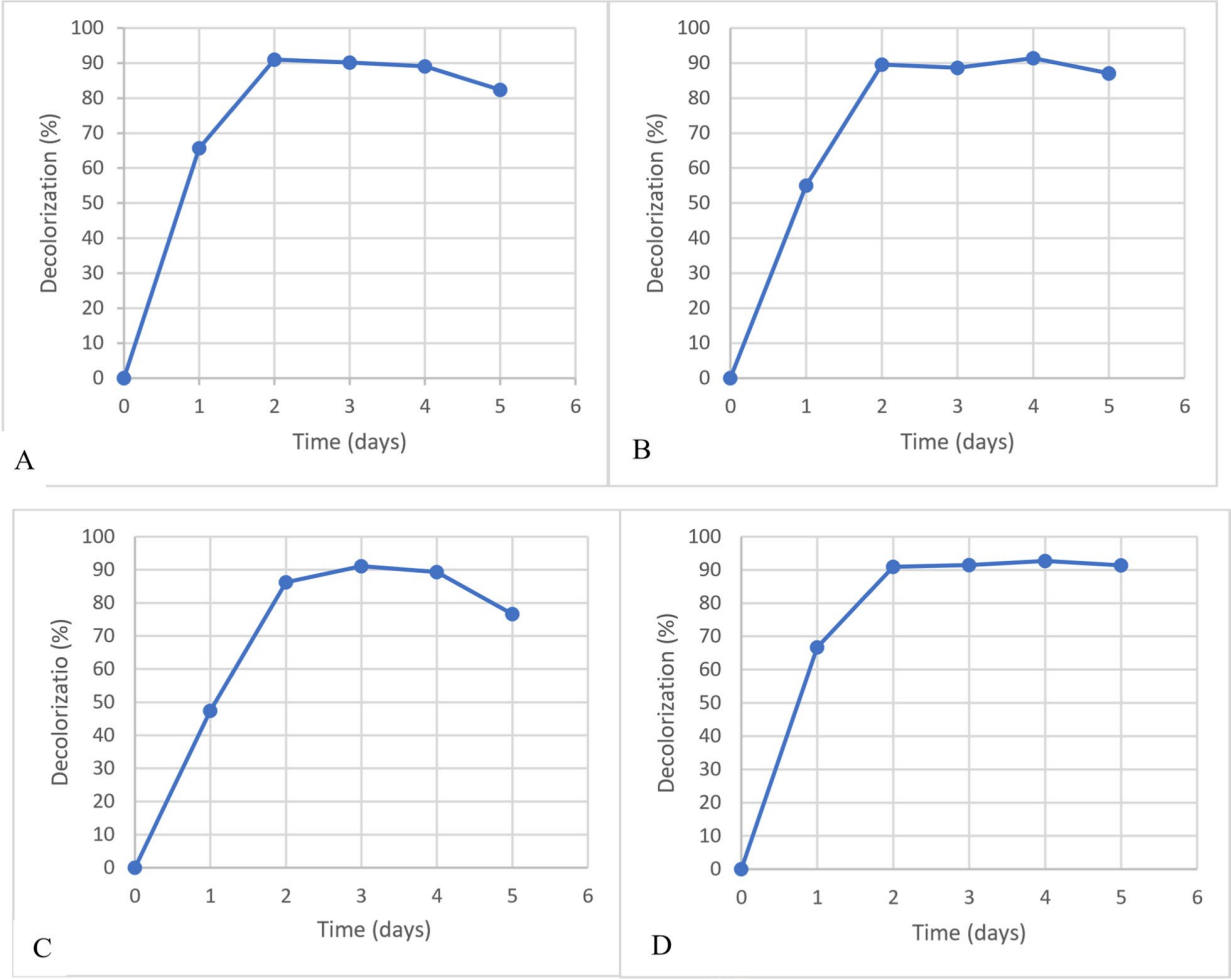
Decolorization of MG of concentration 40 µg/ml by Robiki sample at different conditions: **A** Aerobic, **B** Aerobic and addition of glucose 1%, **C** Anerobic, **D** Anerobic and addition of glucose 1%

The bacteria from Robiki sample were isolated and purified to be inoculated in tubes containing basal mineral media and malachite green of the same concentration to measure absorbance. After 48 h of incubation. MG is difficult to remove from aqueous solutions due to its characteristics, and it is also toxic to major microbes. Most bioremediation research focuses on adsorption. It was found that the decolorization percentage ranged from 6.8–34.3% for aerobic cultures and about 71.1% for anaerobic cultures, indicating that bacterial isolate of Robiki sample used malachite green dye as a carbon source from aqueous solutions aerobic and anaerobic. However, its degradation result in anaerobic condition was more effective. Degradation of MG anaerobically was preferred because it ends up with nontoxic compounds. (Nordlund et al., [17]). Decolorization of MG was more than 92.7% under anaerobic conditions and 89.1% under aerobic conditions for bacteria isolated from Robiki sample (Fig. 1). Adding glucose to the culture provides a source of energy and enhances microbial growth and consortia degradation performance [21].

### Simultaneous decolorization and energy production

**Fig. 2:**
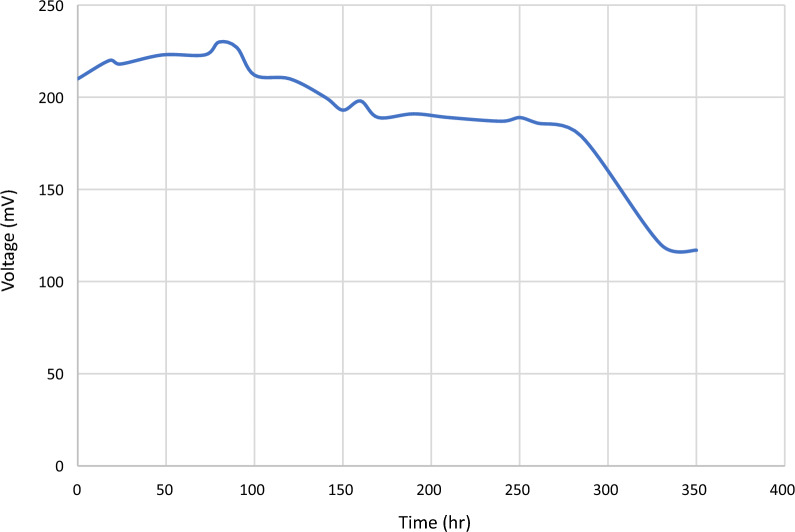
Voltage production in MFC in the presence of 40 ug/ml MG

**Fig. 3:**
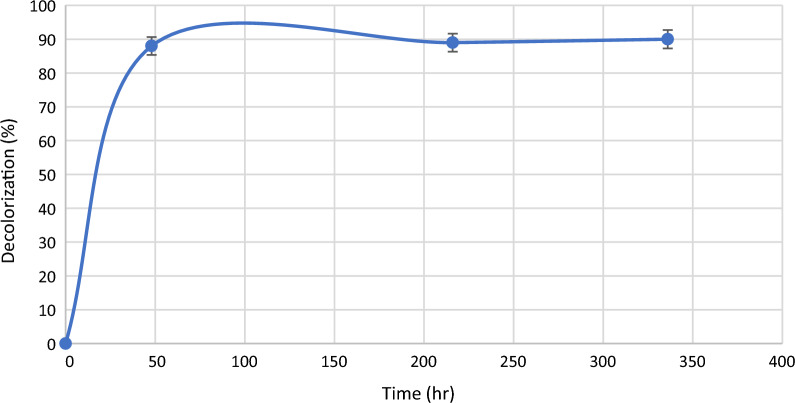
Decolorization profile of MG in the MFC

Robiki sample was used in MFC to test its potential for both decolorization and electricity production. The results in Figs. 2, 3 represent the electricity production and decolorization profile of malachite green in MFC over 14 days. The obtained results indicate an increase in decolorization over time, while electricity production showed a decrease in mV over time. The average output voltage was 195.76 mV. It was previously reported that competition between decolonization and electricity production can take place [11]. For bioelectricity generation, MG should be a more electrochemically active electron shuttle. However, such capacities of redox mediators for MG rapidly decreased, due to the formation of non-quasi reversible conversion of MG to less electrochemically active chemicals (e.g., leuco form of MG or related intermediates) may be occurring [4] The presence of intermediate metabolites can also act as electron shuttle compounds in MFCs which helps in accelerating the degradation and electricity production [20].

**Fig. 4:**
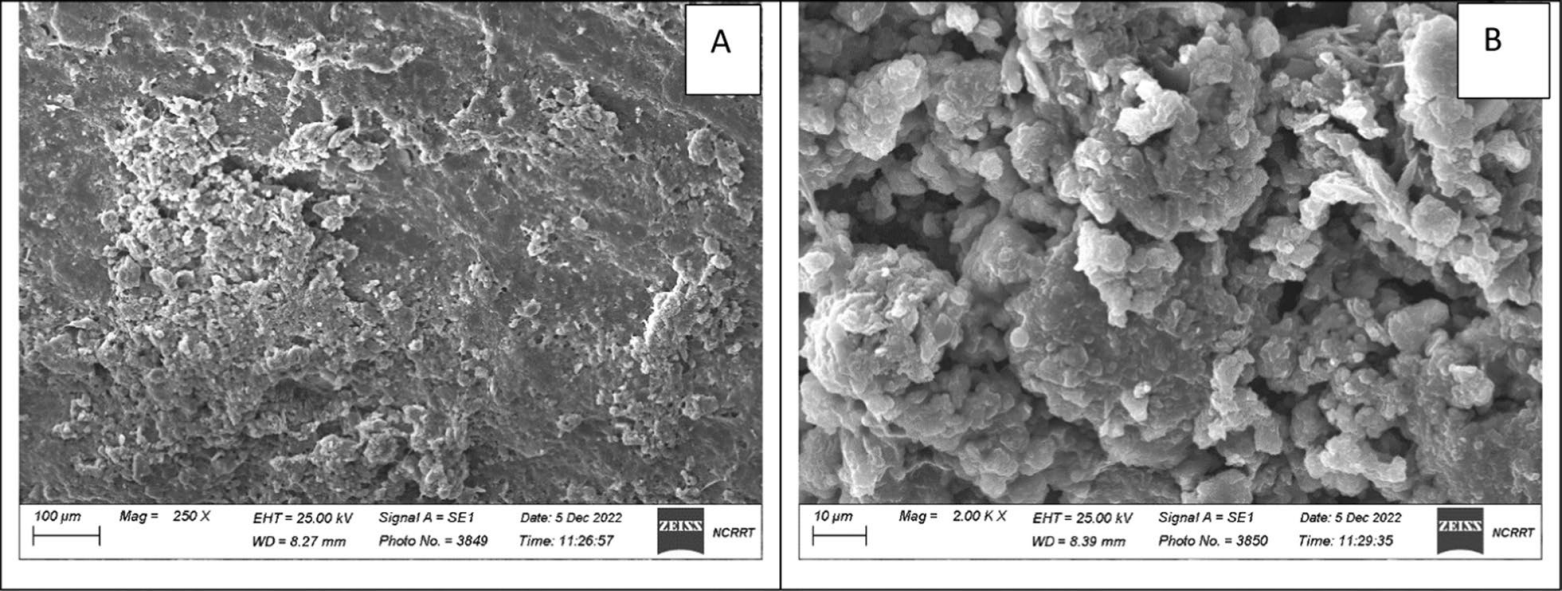
SEM images of biofilm formation on anodic electrode at magnification 250x (**A**), at magnification 2000x (**B**). The images shows bacterial growth on the surface of the anode at the end of the MFC operation

**Fig. 5:**
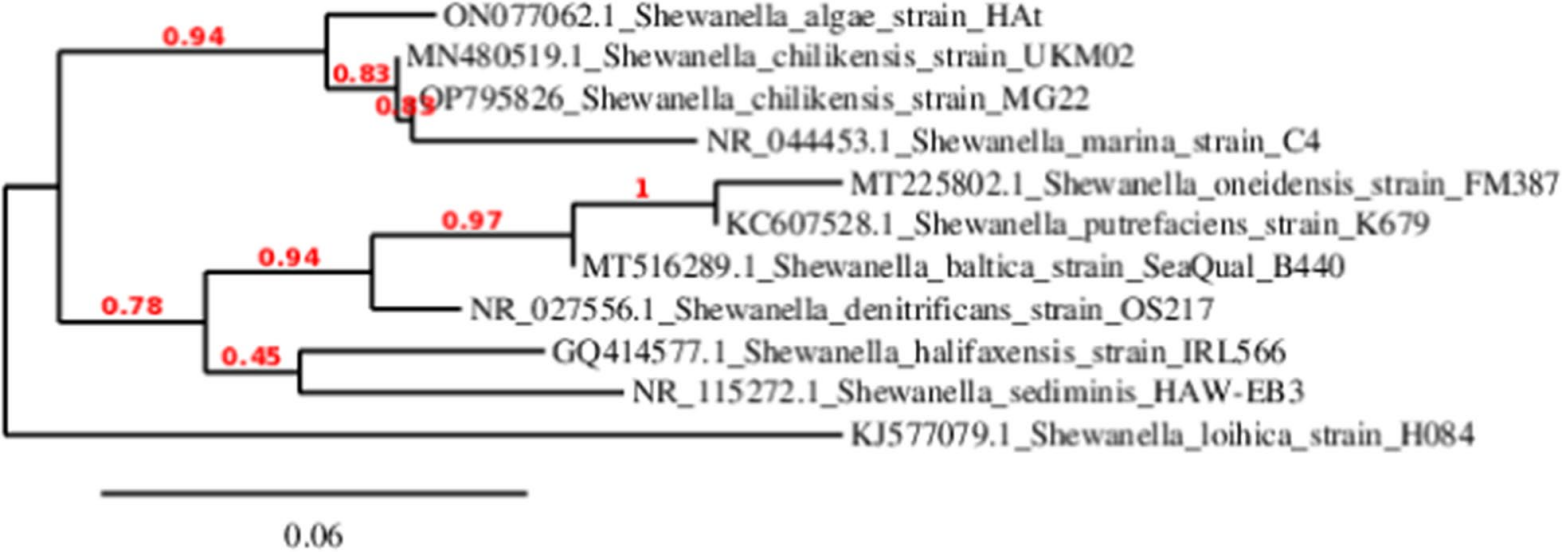
16S rRNA phylogenetic identification of the predominant malachite green decolorizing isolate

At the end of the MFC operation, the biofilm grown and attached to the anode was captured using scanning electron microscopy. The images in Fig. 4 represent the biofilm attached to the anode at two magnifications. Biofilm formation is an important aspect of MFC operation, it’s a mechanism by which electrogenic bacteria transfer electrons from within the cell to the electrode surface. Different bacterial genera are known to transfer electrons via direct electron transfer (DET), the most famous electrogenic bacteria belongs to the *Shewanellaceae* family. In the present study, the predominant bacterium that was responsible for MG decolorization was identified using 16S rRNA phylogenetic identification as *Shewanella chilikensis* strain MG22 (Accession no. OP795826) as shown in Fig. 5. The generated tree shows that the closest relatedness of the bacteria in the present study was to *Shewanella chilikensis*, *Shewanella algae,* and *Shewanella algae* by 94%. On the other hand, other strains such as *Shewanella onedensis, Shewanella putrificans, Shewanella baltica, Shewanella denitrificans, *etc*.* are distant. *Shewanella chilikensis* was reported as alkaliphilic gamma proteobacteria [29]. Based on thermodynamics, alkaliphiles (at pH 10) possess anodic potential of − 0.609 Vs. normal hydrogen electrode (NHE), while that under practical conditions (pH7), the anode potential is -0.291 Vs. NHE [6]. This suggests that in our study, the anode potential is expected to lie in between those two values since the predominant microorganism is an alkaliphile while the anolyte in the anodic compartment was adjusted at pH7. In addition to the enzymes present in the outer membrane of Shewanella genus that contribute to stress response towards different extreme conditions [35], *Shewanella chilikensis* has strong adhesion to surfaces as reported by Tuck et al. [32] which facilitates electron transfer via DET pathway from the cells to the anode. As far as the authors know, this is the first time *Shewanella chilikensis* is mentioned as an electroactive bacterium, however, it was mentioned to possess electroactivity and was responsible for metal surface corrosion by initially forming a biofilm [24]. The lack of sufficient information of this microorganism prompts more research in the fields of electrochemistry and bioremediation (Additional file 1).

### Re-use of MG-containing water in MFC and its sterilization

**Fig. 6:**
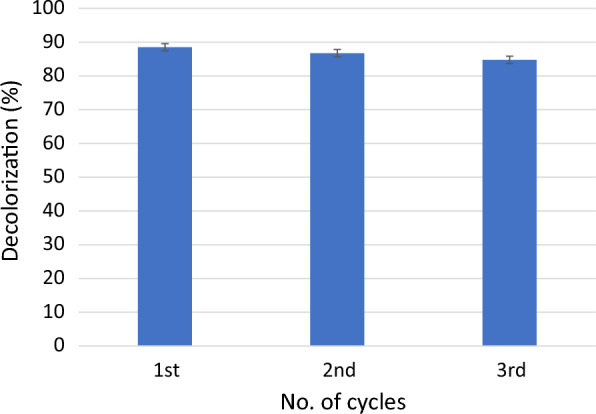
Malachite green removal in MFC for 3 consecutive cycles

**Fig.7:**
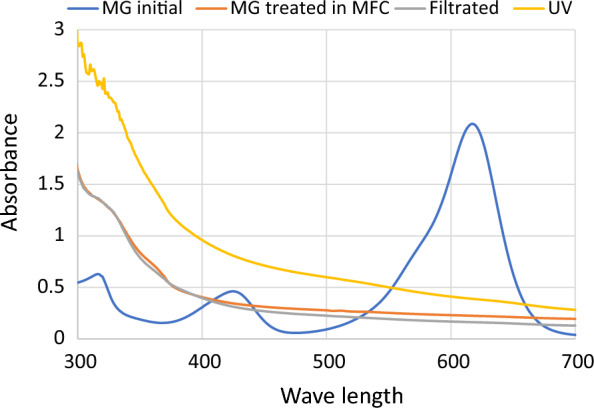
UV–Visible spectrum for treated aquaculture water after UV sterilization and filter sterilization

Since RAS depends on recirculation of aquaculture water in a closed system, we tested MFC efficiency. In the present study, we tested the degradation of malachite green for 3 consecutive cycles. The result shown in Fig. 6 shows that the decolorization is almost the same for 3 cycles. To ensure that treated wastewater can be fed to the aquaculture system again, the treated water was sterilized using a UV lamp at 254 nm and filter sterilization. The results show that although bacterial growth was not detected in both UV and filter-sterilized water (Table 2), the color of UV-exposed water was darker than the one that was filter sterilized. This is evident by the increase in the spectrum for UV-exposed cells as compared to water before treatment (Fig. 7). It also shows that the filter-sterilized water showed less color intensity which indicates that the water quality can be improved with sterilization. Although UV sterilization is considered an efficient method to clarify and disinfect water in aquacultures [12, 14], it was not reported for malachite green containing water. In the present study, the residual brownish color obtained after UV exposure was resembled another study that used ionizing radiation for treating colored water where it was suspected to have undergone polymerization of the degradation by-products (El-Kenawy and Gomaa, in press) which affects the water quality and aesthetic nature. Therefore, it is recommended to use UV to get rid of microbial growth from aquaculture and use filter sterilization to remove residues of color if MG is added.

**Table 2:** Bacterial count before and after sterilization

Sample	Count (cfu)
MG after treatment in MFC	12 × 10^6^
Filter sterilization	ND
UV sterilization	ND

ND indicates no detected colonies on LB agar plates

## Conclusion

The present study demonstrates for the first time *Shewanella chilikensis* as an electroactive bacterium capable of decolorizing dyes and producing electricity. Its ability to form anodic biofilm and repetitive MG decolorization in MFC system make it very suitable for use in Recirculating aquaculture (RAS) set ups in countries that still depend on MG in aquacultures. RAS doesn’t just address sustainable development goal (SDG) 6 which ensure availability and management of sustainable water, but it also addresses SDG2 since it provides an abundance of fish as food supply to achieve zero hunger. To achieve this, the quality of recirculating water must be maintained. A recirculating system filters and cleans the water to ensure water quality and limit water use. We believe that integrating an MFC unit equipped with a filter sterilization unit in RAS would ensure non-hazardous aquaculture water. This system is very promising and very applicable in underprivileged areas. More work will be followed to establish an upscaled system that can be used as large-scale RAS.

## Supplementary Information

**Additional file 1: Figure S1.**. Decolorization on plates. A: Robiki sample degrading MG of concentration 40 µg/ml. B: Mud sample bioaccumulate MG of concentration 40 µg/ml.

## Data Availability

The data and material will be available upon request.
