# Supplementary material for: Shewanella chilikensis MG22 isolated from tannery site for malachite green decolorization in microbial fuel cell: a proposed solution for recirculating aquaculture system (RAS)
Source: Microb Cell Fact. 2023 Aug 1;22:142. doi: 10.1186/s12934-023-02152-9 (PMC10394906; doi:10.1186/s12934-023-02152-9)
Supplement: Supplementary file 1 — Additional file 1: Figure S1.. Decolorization on plates. A: Robiki sample degrading MG of concentration 40 µg/ml. B: Mud sample bioaccumulate MG of concentration 40 µg/ml. [file 12934_2023_2152_MOESM1_ESM.docx]

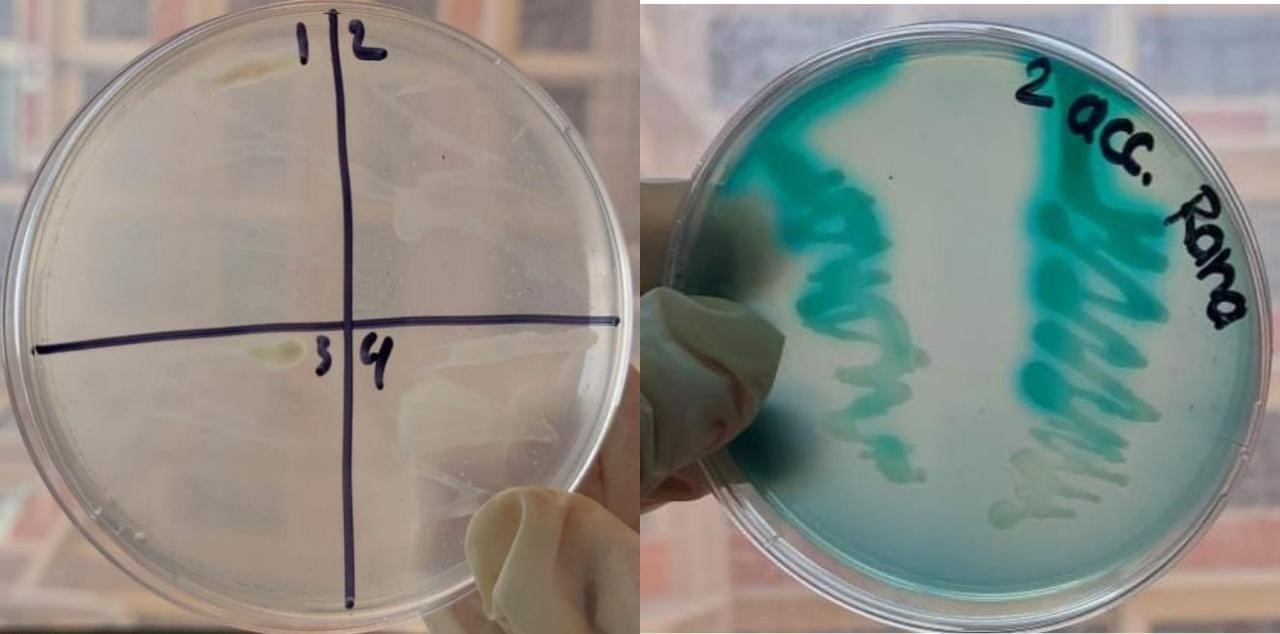


A

B

S1: Decolorization on plates. A: Robiki sample degrading MG of concentration 40 µg/ml. B: Mud sample bioaccumulate MG of concentration 40 µg/ml.
